# Hepatic transcriptome profile of sheep (*Ovis aries*) in response to overgrazing: novel genes and pathways revealed

**DOI:** 10.1186/s12863-019-0760-x

**Published:** 2019-07-04

**Authors:** Weibo Ren, Warwick Badgery, Yong Ding, Huiqin Guo, Yang Gao, Jize Zhang

**Affiliations:** 1 Key Laboratory of Forage Grass, Ministry of Agriculture, Institute of Grassland Research, Chinese Academy of Agricultural Sciences, Hohhot, 010010 Inner Mongolia China; 2 NSW Department of Primary Industries, Orange Agricultural Institute, Orange, NSW 2800 Australia; 3 College of Life Sciences, Inner Mongolia Agricultural University, Hohhot, 010019 Inner Mongolia China; 4 College of Animal Science and Technology, Jilin Agricultural University, Changchun, 130018 Jilin China

**Keywords:** Overgrazing, Liver, Transcriptome, RNA-Seq, Sheep

## Abstract

**Background:**

Overgrazing is a major factor that causes steppe degradation in Inner Mongolian, resulting in extensive ecosystem damage. Scarcity of grass means sheep are smaller and therefore mutton and cashmere production is greatly reduced, which has resulted in massive annual economic losses. Liver is the primary metabolic organ in mammals. It is also the key source of energy supply and detoxification of metabolites in animals, has a close relationship with animal growth. However, investigations on the responses of sheep induced by consequence of overgrazing, particularly those relating to liver-related molecular mechanisms and related metabolic pathways, remain elusive.

**Results:**

The body weight daily gain of sheep, immune organ indices (liver and spleen), and serum parameters related to immune response, protein synthesis and energy supply (IgG, albumin, glucose and non-esterified fatty acid) were significantly lower in the overgrazing group. Other serum parameters including alanine aminotransferase, aspartate aminotransferase, alkaline phosphatase, total bilirubin, blood urea nitrogen and interleukin-6 were significantly higher in the overgrazing group. For the RNA-Seq results, we identified approximately 50 differentially expressed genes, of which half of were up-regulated and the other half were down-regulated (overgrazing group versus light grazing group). Bioinformatics analysis identified two enriched KEGG pathways including peroxisome proliferator-activated receptor (PPAR) signaling pathway (related to lipolysis) and ECM-receptor interaction (related to liver injury and apoptosis). Additionally, several of the down-regulated genes were related to detoxification and immune response.

**Conclusions:**

Overall, based on the high-throughput RNA sequencing profile integrated with the results of serum biochemical analyses, consequences of lower forage availability and quality under overgrazing condition induced altered expression levels of genes participating in energy metabolism (particularly lipid metabolism) and detoxification and immune responses, causing lipolysis and impaired health status, which might be key reasons for the reduced growth performance of sheep. This investigation provides a novel foundation for the development of sheep hepatic gene interactive networks that are a response to the degraded forage availability under overgrazing condition.

**Electronic supplementary material:**

The online version of this article (10.1186/s12863-019-0760-x) contains supplementary material, which is available to authorized users.

## Background

In China, the steppes of Inner Mongolian are mainly utilized in mutton, milk and cashmere production [1] In recent decades, overgrazing has severely damaged this natural grassland, which threatens the normal morphology of forages (lower forage production and imbalanced nutrients composition) and then reduces the individual animal growth performance [2–4]. Increases in stocking rate (SR) have induced grassland plants to accumulate volatile organic compounds as well as altered their morphology [5]. Multi-year investigations have shown that overgrazing significantly decreases body weight gain in sheep during grazing [6, 7].

The mammalian liver serves as the metabolic center and largely contribute to the in vivo metabolism of a wide range of nutrients, such as carbohydrates, proteins, and lipids. In addition, the liver also functions in the generation of immune and inflammatory responses, as well as in xenobiotics clearance [8, 9]. Thus, because the liver is an important, metabolically active organ, hepatic response has been examined to explore the regulatory mechanisms of animal growth [10]. Previous studies have examined the impact of overgrazing on grassland productivity and animal behavior [11, 12]. However, to date, little quantitative data are available on the growth-related hepatic responses of sheep due to consequences of overgrazing (especially lower forage availability and quality), which greatly interferes with defining an optimal grazing intensity (GI) and improving animal production.

Transcriptome analysis is widely applied to study comparative gene expression [13, 14]. The advent of next-generation sequencing technologies has improved and hastened expression profiling at the genome-level, providing extensive genetic information [15, 16]. Numerous studies show that marked alterations occur in hepatic transcriptome profiles of ruminant animals due to changes in environmental factors or dietary composition, particularly novel genes and pathways related to nutrient metabolism (glucogenesis, fatty acid oxidation, amino acids metabolism), cell growth and transcriptional and translational regulation (DNA replication and transcription) [17, 18]. Therefore, the goals of the present study were to perform transcriptome sequencing of sheep liver to detect differentially expressed genes (DEGs) in response to overgrazing triggered forage degradation, elucidating molecular mechanisms of lower animal growth performance.

## Results

### Effects of overgrazing on forage nutritional contents and sheep growth performance

In the present study, the overgrazing (OG) group exhibited higher crude protein (CP) and acid detergent lignin (ADL) content compared to the light grazing (LG) group (*P* < 0.05). The gross energy and nitrogen free extract (NFE) levels of the OG group were significantly lower (*P* < 0.05) relative to the LG group (Additional file 1: Table S1). No significant differences of neutral detergent fiber (NDF) and acid detergent fiber (ADF) was observed in both groups (Additional file 1: Table S1). For the entire grazing experimental period (90 days), sheep under the OG condition had less daily weight gain (DWG) (*P* < 0.05) and lower carcass weight (*P* < 0.05; Table 1). Additionally, the indices of spleen and liver decreased significantly in the OG sheep (*P* < 0.05; Table 1).

**Table 1:** The effects of overgrazing on sheep growth and immune organ indices

Item	Groups	
	LG	OG
DWG (g/d)	156 ± 36^a^	133 ± 24^b^
Carcass weight cold (kg)	23.2 ± 1.5^a^	21.1 ± 0.9^b^
Index of spleen (%)^1)^	0.46 ± 0.04^a^	0.32 ± 0.07^b^
Index of liver (%)	3.38 ± 0.14^a^	2.95 ± 0.08^b^

Values within a row with different superscript letters represent significant differences at *P* < 0.05. Numbers are expressed as the means ± SD. (DWG, *n* = 12 for LG and *n* = 36 for OG; indices of immune organs, *n* = 6)

^1)^ Immune organ indices were computed as the ration of organ weight to body weight

*DWG* Daily weight gain, *LG* Light grazing, *OG* Overgrazing

### Effects of overgrazing on serum parameters of sheep

Based on the results of serum biochemical indices, inflammatory responses and immune responses (Table 2), degraded forage availability and quality under OG condition significantly affected these parameters. Alanine aminotransferase (ALT), aspartate aminotransferase (AST) and alkaline phosphatase (ALP) activities, total bilirubin (TBIL), serum blood urea nitrogen (BUN) and interleukin-6 (IL-6) concentrations significantly increased in the OG group compared to the LG group (*P* < 0.05). By contrast, OG sheep had lower concentrations of albumin (ALB), glucose (GLU), non-esterified fatty acid (NEFA) and immunoglobulin G (IgG) than those of the LG sheep (*P* < 0.05).

**Table 2:** The effect of overgrazing on sheep serum biochemical parameters

Item	Groups	
	LG	OG
ALT (IU/L)	27.70 ± 9.13^b^	38.18 ± 6.36^a^
AST (IU/L)	129.95 ± 12.04^b^	146.12 ± 12.65^a^
ALP (U/L)	206.83 ± 14.87^b^	273.51 ± 24.52^a^
ALB (g/L)	30.65 ± 2.59^a^	25.12 ± 3.57^b^
TBIL (mmol/L)	4.29 ± 0.78^b^	6.13 ± 1.04^a^
BUN (mmol/L)	8.92 ± 0.45^b^	10.90 ± 1.19^a^
GLU (mmol/L)	5.86 ± 1.11^a^	4.53 ± 0.94^b^
NEFA (mmol/L)	0.59 ± 0.03^a^	0.41 ± 0.05^b^
IL-6 (pg/mL)	86.08 ± 20.93^b^	211.34 ± 10.27^a^
IgG (g/L)	18.74 ± 1.87^a^	13.26 ± 1.55^b^

Values within a column with different superscript letters represent significant differences at *P* < 0.05. Numbers are expressed as the means ± SD. (*n* = 6)

*LG* Light grazing, *OG* Overgrazing

*ALT* Alanine aminotransferase, *AST* Aspartate aminotransferase, *ALP* Alkaline phosphatase, *ALB* Albumin, *TBIL* Total bilirubin, *BUN* Blood urea nitrogen, *GLU* Glucose, *NEFA* Non-esterified fatty acid, *IL* Interleukin, *IgG*, Immunoglobulin G

### Overall assessment of mapping statistics

**Fig. 1:**
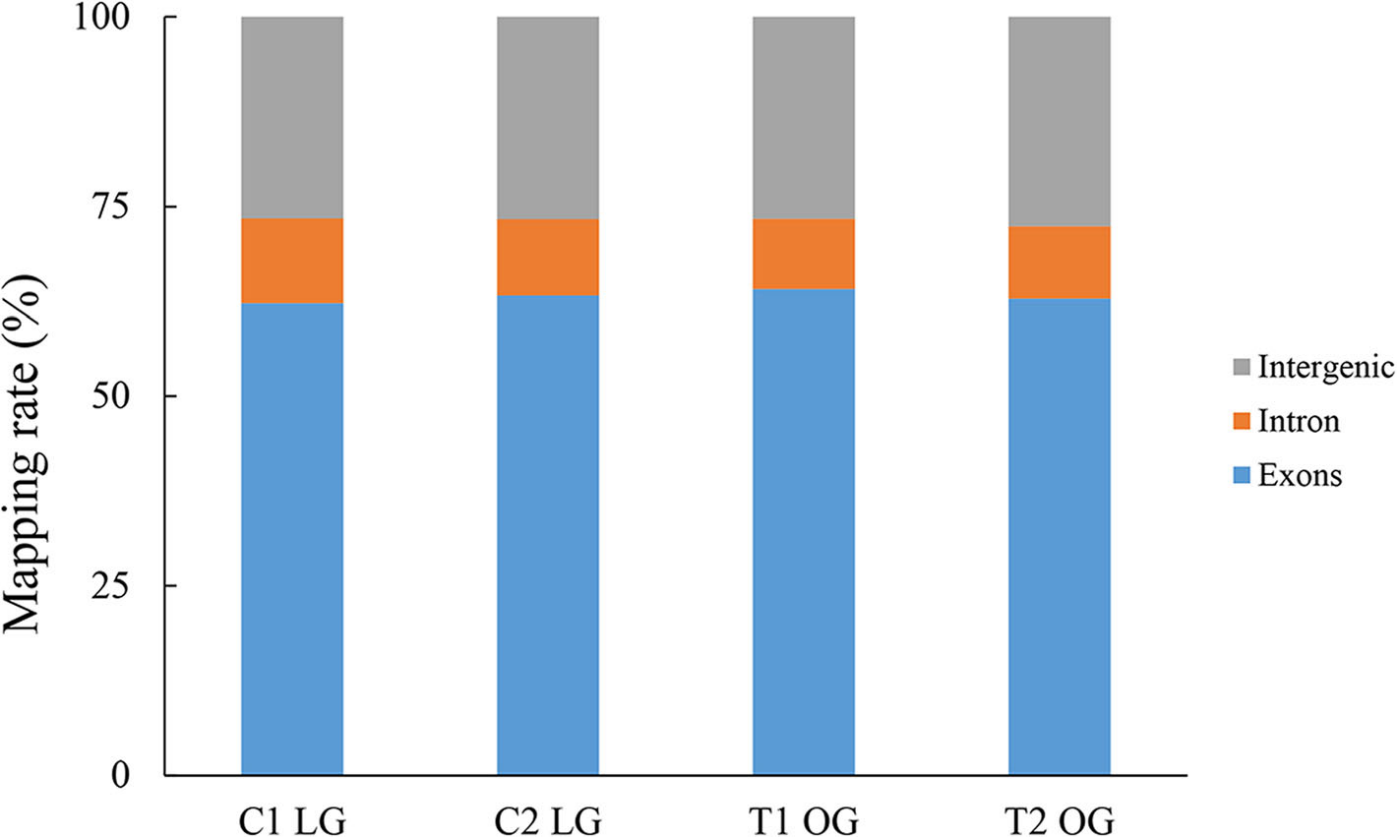
The percentage of reads mapped to exonic, intronic, and intergenic regions. LG, light grazing; OG, overgrazing. C1 and C2, pooled biological replicates in LG group. T1 and T2, pooled biological replicates in OG group

The RNA-Seq of the four pooled liver samples generated a total of 141.8 million raw paired-end reads. Upon quality filtering, approximately 6.4 Gb remained as high-quality sequence data in each sample. Using TopHat2 software, we were able to map more than 74.08% of clean reads per sample to the reference genome, with 71.66–72.46% of the reads depicting unique alignment. The alignment summary of all samples is shown in Table 3. The majority of the reads (62.20–64.09%) were mapped to annotated exons, whereas 26.58–27.60% were mapped to the large regions of intergenic sequences, and only 9.29–11.22% were localized to intronic regions (Fig. 1). All reads were submitted to the website of the National Center for Biotechnology Information Sequence Read Archive site, with accession number SRP149290.

**Table 3:** Summary statistics of sequence quality and alignment information of four pooled liver samples from two groups

Sample group	C1 LG	C2 LG	T1 OG	T2 OG
Raw reads	40,726,399	35,081,248	30,387,651	35,584,097
Clean reads	37,187,459	31,030,493	25,747,921	32,929,559
Q30 (%)	85.06%	85.08%	85.13%	85.12%
GC Content (%)	49.50%	49.78%	49.91%	50.50%
Mapped reads	27,714,503	23,061,884	19,281,470	24,449,321
Uniquely mapped reads	26,783,720	22,292,733	18,655,917	23,595,965
Mapping ratio (%)	74.53%	74.32%	74.89%	74.08%

*LG* Light grazing, *OG* Overgrazing

### Gene differential expression analysis

**Fig. 2:**
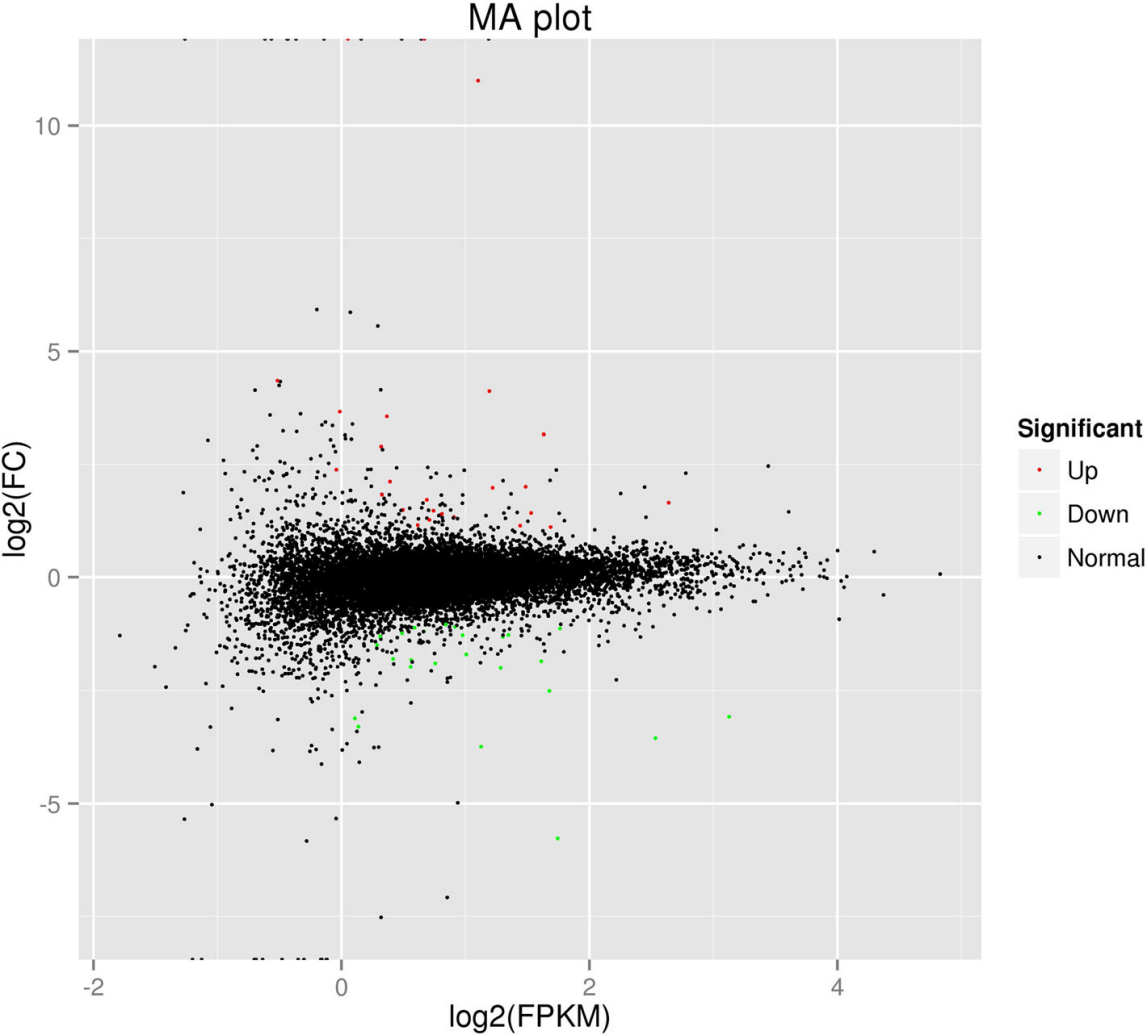
MA plot. Red dots (Up) represent significantly up-regulated genes (*P* < 0.05, fold change ≥2); green dots (Down) indicate significantly down-regulated genes (*P* < 0.05, fold change ≤0.5); black dots (Normal) show insignificantly differentially expressed genes

RNA-Seq results from the LG and OG groups identified 12,860 genes when annotated to sheep reference genome (Additional file 2: Table S2 and Fig. 2). Using the criteria of log2 ratio ≥ 1 and a *P*-value < 0.05, a total of 50 genes were identified as DEGs (OG group versus LG group) (Table 4). Furthermore, comparative analysis of the LG and OG groups indicated equal numbers of down- and up-regulated genes. More importantly, several of the down-regulated genes were related to detoxification (*UGT2B31*, *GLYAT*, *GSTA1*, *RHBG*) and immune response (*DDX58*, *LRRC40*, *MX1*, *IFI27L2*) (Table 4), which played vital roles in sheep health and growth performance.

**Table 4:** Differentially expressed genes in overgrazing group compared to light grazing group

Ensemble ID	Gene symbol	Annotation	log2FC
Down-regulated genes			Down
ENSOARG00000008430	SCN2B	Sodium channel subunit beta-2	−5.8
ENSOARG00000009284	UGT2B31	UDP-glucuronosyltransferase 2B31	−3.7
ENSOARG00000014413	IFI27L2	Interferon alpha-inducible protein 27-like protein 2	−3.6
ENSOARG00000018317	COL4A5	Collagen alpha-5(IV) chain	−3.3
ENSOARG00000013977	ROCK2	Rho-associated protein kinase 2	−3.1
ENSOARG00000006050	ORM1	Alpha-1-acid glycoprotein	−3.1
ENSOARG00000011261	GLYAT	Glycine N-acyltransferase	−2.5
ENSOARG00000018851	CEP290	Centrosomal protein of 290 kDa	− 2.0
ENSOARG00000005827	C1ORF85	Lysosomal protein NCU-G1	−2.0
ENSOARG00000017176	TCTN3	Tectonic-3	−1.9
ENSOARG00000011449	PRLR	Prolactin receptor	−1.9
ENSOARG00000005587	EXT1	Exostosin-1	−1.8
ENSOARG00000020058	THBS1	Thrombospondin-1	−1.8
ENSOARG00000014902	GSTA1	Glutathione S-transferase A1, N-terminally processed	−1.7
ENSOARG00000014731	DDX58	Probable ATP-dependent RNA helicase DDX58	−1.5
ENSOARG00000005951	RHBG	Ammonium transporter Rh type B	−1.3
ENSOARG00000017047	ALDH1L1	Cytosolic 10-formyltetrahydrofolate dehydrogenase	−1.3
ENSOARG00000011814	LRRC40	Leucine-rich repeat-containing protein 40	−1.3
ENSOARG00000005886	PFKFB1	Fructose-2,6-bisphosphatase	−1.3
Sheep_newGene_2200			−1.2
ENSOARG00000010283	MX1	Interferon-induced GTP-binding protein Mx1, N-terminally processed	−1.2
ENSOARG00000012673	RCAN1	Calcipressin-1	−1.1
ENSOARG00000017875	COL8A1	Vastatin	−1.1
ENSOARG00000010155	RNF144B	E3 ubiquitin-protein ligase RNF144B	−1.1
ENSOARG00000019483	FOS	Proto-oncogene c-Fos	−1.0
Up-regulated genes			Up
Sheep_newGene_75			Inf
Sheep_newGene_7857			Inf
ENSOARG00000011401	RXRG	Retinoic acid receptor RXR-gamma	11.0
Sheep_newGene_4617			4.4
Sheep_newGene_7585	SULT3A1	Amine sulfotransferase	4.1
ENSOARG00000000581	LRRC31	Leucine-rich repeat-containing protein 31	3.7
ENSOARG00000003294	CYP4A6	Cytochrome P450 4A6	3.6
ENSOARG00000009458	AQP7	Aquaporin-7	3.2
Sheep_newGene_1150			2.9
ENSOARG00000013615	FSTL1	Follistatin-related protein 1	2.4
ENSOARG00000010334	KLKB1	Plasma kallikrein light chain	2.1
ENSOARG00000010449	DHDH	Dihydrodiol dehydrogenase 3	2.0
ENSOARG00000005941	TNC	Tenascin	2.0
Sheep_newGene_3			1.8
ENSOARG00000009239	BCL3	B-cell lymphoma 3 protein	1.7
ENSOARG00000004859	HOP	Homeodomain-only protein	1.7
ENSOARG00000004234	SLC45A3	Solute carrier family 45 member 3	1.5
ENSOARG00000000804	POC1B	POC1 centriolar protein homolog B	1.5
ENSOARG00000011986	RPL10A	60S ribosomal protein L10a	1.4
ENSOARG00000013534	MYOC	Myocilin, C-terminal fragment	1.4
ENSOARG00000010264	PDLIM2	PDZ and LIM domain protein 2	1.3
Sheep_newGene_8346	CFH	Complement factor H	1.3
ENSOARG00000000813	TECPR1	Tectonin beta-propeller repeat-containing protein 1	1.2
ENSOARG00000020020	TUBA4A	Tubulin alpha-4A chain	1.1
ENSOARG00000017743	SLC51B	Organic solute transporter subunit beta	1.1

*FC* Fold change, Inf, positive infinity, it indicates that the gene is only expressed in the overgrazing group

### Confirmation of RNA-Seq experiment

**Fig. 3:**
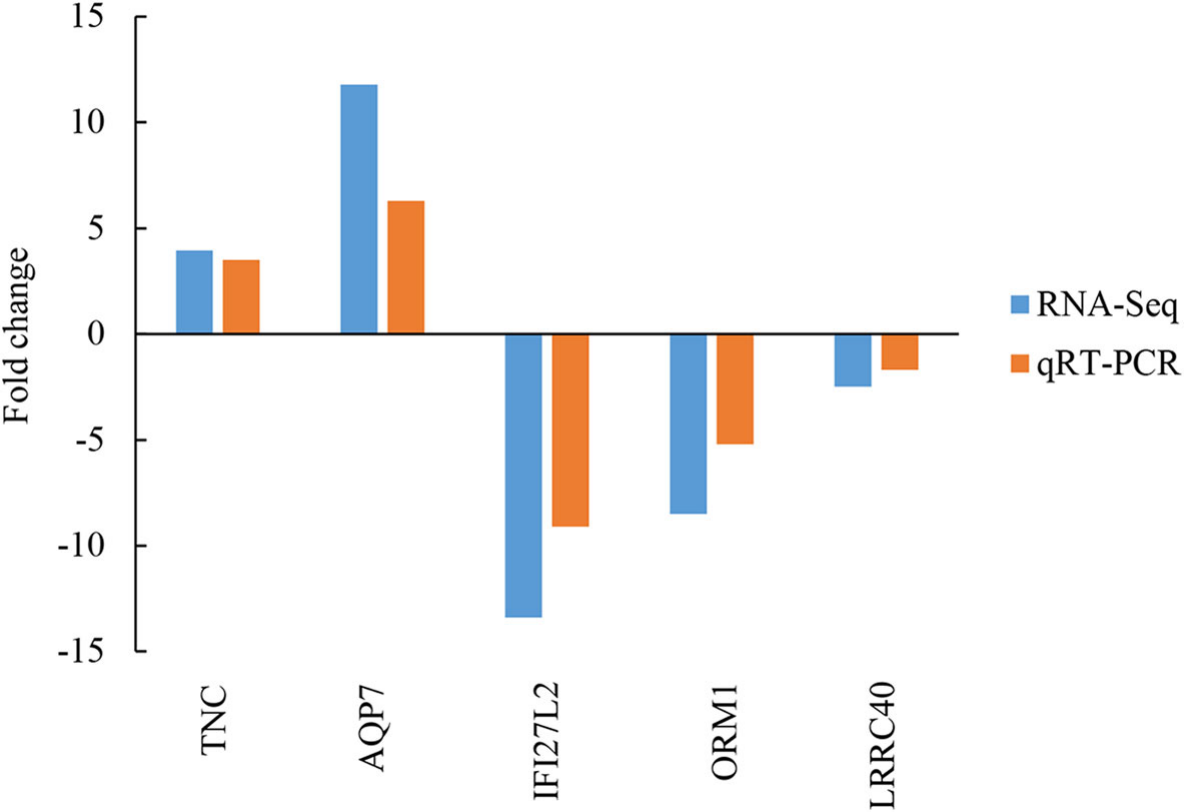
qRT-PCR validation of differentially expressed genes in sheep livers. β-actin (*ACTB*) was used as an internal reference, and the data are expressed as fold-change (*n* = 6 per group)

To evaluate the reliability of our RNA-Seq results, we performed qPCR using aliquots obtained from the non-pooled RNA samples. Five randomly selected protein-coding genes (*TNC*, *AQP7*, *IFI27L2*, *ORM1*, and *LRRC40*) were used in this assay. We observed that the RNA sequencing results coincided with the qPCR findings (Fig. 3), thereby confirming the reliability of our RNA-Seq expression data.

### GO annotations and pathway analysis of differentially expressed genes

**Fig. 4:**
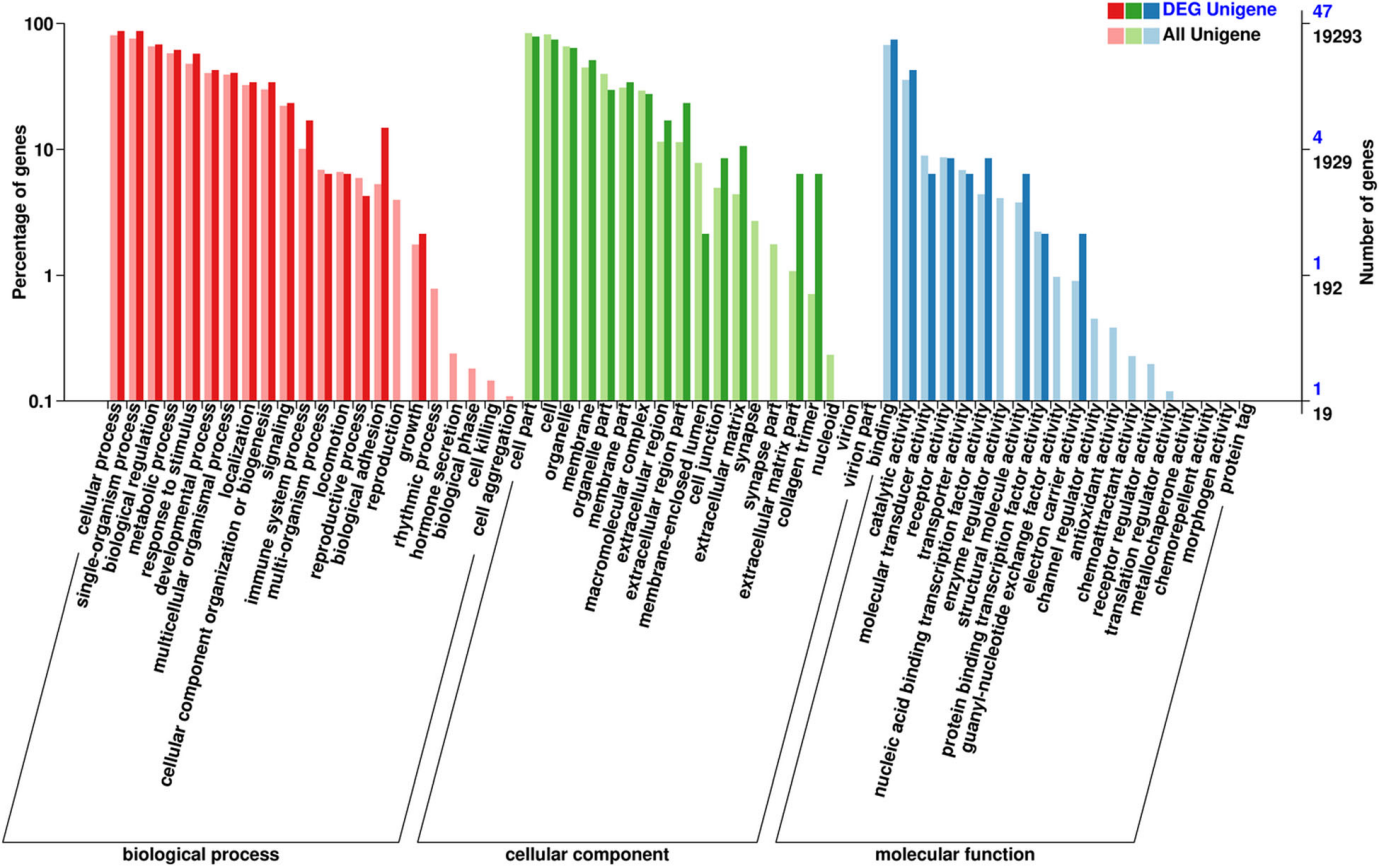
Gene ontology (GO) annotation of unigenes and differentially expressed genes (DEGs) in light grazing sheep as well as overgrazing sheep

Gene ontology (GO) was employed for the functional annotation of DEGs in our two study groups. For the category of biological processes, cellular processes, single-organism processes, biological regulation (immune responses) and metabolic processes (energy metabolism) were the most abundant groups (Fig. 4), thereby suggesting that the impaired forage quantity and quality induce changes in sheep health and growth. In the category of cellular components, the subcategories of cell part, cell, organelle, and membrane were ranked top 3 (Fig. 4). For the molecular functional group, the subcategories of binding (nucleic-acid binding) represented the largest term, followed by catalytic and structural molecular activity. KEGG pathway analysis identified two highly enriched pathways, namely, the peroxisome proliferator-activated receptor (PPAR) signaling pathway (*P* = 0.0060) as well as the ECM-receptor interactions (*P* = 0.0125) (Table 5).

**Table 5:** Pathways that are significantly enriched with differentially expressed genes

Pathway name	Enriched genes	*P*-value
PPAR signaling pathway	RXRG, CYP4A6, AQP7	0.0060
ECM-receptor interaction	COL4A5, THBS1, TNC	0.0125

*RXGH* Retinoic acid receptor RXR-gamma, *CYP4A6* Cytochrome P450 4A6; AQP7, aquaporin-7; *COL4A5* Collagen alpha-5(IV) chain, *THBS1* Thrombospondin-1, *TNC* Tenascin

## Discussion

The liver is the major organ in animals that is involved in the metabolism of lipids, nitrogen compounds, as well as energy [19]. Alterations in nutrients metabolic pathways are closely related to animal growth performance and health [20]. In this study, high-throughput transcriptomic analysis integrated with serum parameter analyzes were conducted to elucidate the underlying molecular mechanisms in growth reduced sheep livers in response to consequences of OG. Like previous findings, we observed a negative correlation between elevated GI and growth performance among sheep [6].

The LG and OG groups exhibited differences in the transcript expression of 50 genes. In a previous study, additional energy that would be utilized for animal physical activity are necessary with the increased GI, which in turn reduces the available energy needed for growth and production [3]. Our findings agree with those of Lin et al. (2011) [3], in that we observed changes in genes expression and serum components in relation to energy metabolism because of the lower forage availability. DEGs involved in the PPAR signaling pathway were identified in this study function in lipid metabolism, including aquaporin-7 (*AQP7*), cytochrome P450 4A6 (*CYP4A6*) and retinoic acid receptor RXR-gamma (*RXRG*). These DEGs that are related to lipid metabolism possibly contribute to lipolysis, which occurs in the liver, to provide energy and structural stability to the body [21].

AQPs are intrinsic membrane proteins that form channels in epithelial and endothelial cells [22]. *AQP7* apparently regulates the efflux of lipolysis-derived glycerol from adipocytes, which are essential in the maintenance of normal adiposity [22]. A previous study had shown that *AQP7* is up-regulated by fasting, low insulin and PPARα and altered expression may be related to lipid metabolism disorders [23]. Similar to these functions, *AQP7* expression was significantly higher in the OG group compared to the LG group, possibly due to the significant decreased in herbage allowance in heavy grazed areas [3]. The transcriptional activation of *CYP4A6* in response to peroxisome proliferators can be mediated by PPARα, and the peroxisome proliferators are accompanied by the induction of enzymes involved in fatty acid oxidation caused by fasting [24]. Therefore, *CYP4A6* might function as an indicator of extra fatty acid oxidation in sheep induced by the shortage of herbage under the OG condition. *RXRG* is an important regulator of fatty acid metabolism and β-oxidation depending on the binding of RXR ligands or PPAR ligands [25]. The up-regulated expression of *RXRG* reflects a metabolic adaption to a higher lipolysis and fatty acid turnover due to their regulatory roles [13]. The elevated expression of *RXRG* in the OG group might reflect similar mechanisms to those provided by Boergesen et al. (2012) and Óvilo et al. (2014) [13, 26] that increases hepatic fatty acid oxidation. In this study, lower herbage availability caused by OG was associated with increased expression of *AQP7*, *CYP4A6* and *RXRG*, inducing lipolysis and fatty acid oxidation, which is consistent with previous research [3]. In addition to the molecular evidence shown in the hepatic transcriptome, serum levels of GLU and NEFA, which are important energy sources, were greatly decreased in the OG sheep. The above combined results indicated that the shortage of forage availability increased sheep physical activity, which in turn reduced the available energy for growth and production under the OG condition.

In addition to lipid metabolism, the liver also serves as the site of detoxification, thereby eliminating endogenous toxic compounds that are generated during metabolism or exogenous xenobiotics [27]. In this study, four DEGs related to detoxification were down-regulated in OG sheep livers. Primarily expressed in the liver, UDP-glucuronosyltransferase 2B31 (*UGT2B31*) is one of the most important xenobiotic metabolizing enzymes that conjugate a wide range of xenobiotics [28]. Glycine N-acyltransferase (*GLYAT*) plays a major role in maintaining hepatic homeostasis and contributes to glycine conjugation of xenobiotics as well as endogenous metabolites that are involved in inhibition fatty acid oxidation in hepatic tissues [29]. The glutathione S-transferases (*GSTs*) pertain to a superfamily of isoenzymes that are involved in the detoxification of xenobiotics and endobiotics, cellular protection from oxidative stress, and regulation of signaling pathways [30]. Glutathione S-transferase A1 (*GSTA1*) is the predominant GST in the liver and participates in the metabolism of lipid peroxidation products [30]. Ammonium transporter Rh type B (*RHBG*) is an ammonia transporter, which is preferentially expressed in the sheep liver for acid-base homeostasis [31]. Genetic deletion of *RHBG* alters basal ammonia excretion in response to an acid load. The down-regulation of these genes indicates impairment of hepatic detoxification in the OG group, which coincides with the observed elevated serum ALT, AST, ALP, TBIL and BUN levels. Impairment of hepatic detoxification in the OG group might be triggered by a shift in energy sources; namely, from carbohydrates to fatty acids and proteins, which may be due to shortage of forage allowance induced excessive physical activity and unequal levels of gross energy and CP levels in the forage. This speculation is consistent with our demonstration in the previous study [19].

In addition to PPAR signaling pathway, ECM-receptor interaction was significantly enriched in liver transcriptome of OG sheep. As reported in previous study, dysregulation of this pathway in hepatic tissue or cell can induce liver injury and apoptosis [32]. Because the ECM plays an important role in maintaining cell structure and function [33]. We observed in liver transcriptome lower expression of collagen gene (collagen alpha-5(IV) chain) (*COL4A5*) and altered expression of thrombospondin-1 (*THBS1*) and tenascin (*TNC*) in the OG group that could lead to disruption of the ECM [32, 34]. The above alterations of transcriptome profile combined with the increased serum level of IL-6, suggests that the imbalanced forage nutrients composition may trigger the pathological change of sheep liver. However, additional determinations are still required on histological evidences such as HE staining, immunohistochemical staining and so on in the future.

The liver is an important immune organ that contributes to protection from bacterial or viral infection and anti-inflammatory reactions [35]. The OG group showed that four immune response-related genes were down-regulated. Probable ATP-dependent RNA helicase DDX58 (*DDX58*), Leucine-rich repeat-containing protein 40 (*LRRC40*), and interferon-induced GTP-binding protein Mx1 (*MX1*) are related to the activation of the innate immune system, thereby driving adaptive immune responses [36–39]. Interferon alpha-inducible protein 27-like protein 2 (*IFI27L2*) is one of a family of cytokines with antiviral function [40]. The down-regulation of these genes implies that the sheep immune system has been suppressed under OG conditions, which increases the likelihood of pathogenic infections and decreases growth performance. This observation coincides with the decreased immune indices and serum IgG levels of the OG sheep. Moreover, an anti- inflammatory reaction-related gene was down-regulated in the OG group. Alpha-1-acid glycoprotein (*ORM1*) possesses some anti-inflammatory and immunomodulatory properties and is primarily expressed in the liver in various species [41]. *ORM1* down-regulation in the OG sheep might lead to chronic stress, which coincides with the higher serum levels of inflammatory cytokines. We propose that the DEGs related to detoxification, immune response and anti-inflammatory reaction altered by degraded forage quality collectively indicated that the health status of sheep was severely impaired under in the OG group, which might be another important reason for the reduced growth performance.

## Conclusion

In summary, the transcriptome analysis has shown that consequences of OG leads to differential abundances of various hepatic genes in sheep. Furthermore, high-throughput RNA sequencing and serum biochemical analysis revealed that lower forage availability and quality induces alterations in energy metabolism (i.e., lipid metabolism) as well as detoxification and immune responses, resulting in lipolysis and impaired health status, ultimately reducing sheep growth. The results of the present study provide a foundation for the development of gene interactive networks that are involved in OG induced side effects as well as nutritional strategies to enhance sheep growth performance under OG conditions.

## Methods

This research was performed at the Institute of Grassland Research, the Chinese Academy of Agricultural Sciences Research Station in the summer 2016. The station is situated in the Xilin River basin of Inner Mongolia Autonomous Region of China (116°32′ E, 44°15′ N). Its natural vegetation is predominated by three grass species: namely, *Leymus chinensis*, *Stipa krylovii*, and *S. grandis*.

### Experimental design and animals

In June 2016, a total of 48 adult Uzhumchin wethers (24-month-old; initial live weight (LW):33.2 ± 3.9 kg) were purchased from Xinshengjia Sheep Breeding company (Xilin Hot, Inner Mongolia, China). The animals were randomly distributed to the LG (4 sheep/plot) and the OG (12 sheep/plot) groups. The experimental site was consisted of 6 total plots (1.33 ha/plot, 3 plots per each grazing group). Therefore, two different stocking rates (SRs) were formed: 3.0 (LG) and 9.0 sheep/ha (OG). The animals grazed continuously in the plots throughout the entire grazing experiment (June 5 to September 3, 90 days). The sheep received treatment for internal parasites at the start of the investigation and were provided water and mineral lick stones ad libitum.

### Sample collection

To determine chemical composition, the forages were pooled each month until completion of the grazing experiment. The procedure of collecting forages was as previously described [42] and details are presented in Additional file 3. Various chemical composition indices of the collected forages were assessed, which included CP, gross energy, NFE, NDF, ADF, and ADL, as previously described [6]. The live weight (LW) of the animals were determined the first and the last day (following 12-h fasting) of the grazing study and used in calculating the mean daily gain. Blood were then collected from two randomly selected sheep from each plot in the two GI groups (*n* = 6 for each group) via venopuncture of the cervical vein and into vacuum tubes. The blood samples were then centrifuged twice at 2000 *g* at 4 °C for 30 min, followed by 400 *g* at 4 °C for 10 min, to separate the sera, which were then stored at − 80 °C until analysis. The sheep used in blood extraction were sacrificed by CO_2_ asphyxiation for liver sample collection using standard procedures approved by the Chinese Academy of Agricultural Sciences (2 sheep per plot, *n* = 6 for each group). Immediately, after exsanguination, the spleen and liver were isolated and weighed and used in the calculation of immune organ indices. For liver transcriptome and qPCR analyses, right away after weighting, 2 grams of each liver sample were washed in ice-cold, sterile PBS, frozen in liquid nitrogen, and then stored at − 80 °C.

### Serum biochemical analyses

For biochemical analysis, serum concentrations of ALT, AST, ALP, ALB, TBIL, BUN, GLU, NEFA, IL-6 and IgG were assessed using a corresponding diagnostic kit (Nanjing Jiancheng Bioengineering Institute, Nanjing, China), following the manufacturer’s instructions.

### RNA isolation and sequencing

The liver samples of the 12 sacrificed sheep (six sheep per group) were respectively ground in liquid nitrogen. Total RNA was extracted from the liver samples using TRIzol® (Invitrogen, USA) following the manufacturer’s instructions. RNA parameters such as purity, quantity, and quality were respectively assessed using a NanoPhotometer spectrophotometer (Implen, USA), Qubit 2.0 Fluorimeter (Life Technologies, USA), and Agilent 2100 Bioanalyzer (Agilent Technologies, USA), which revealed that all samples possessed an RNA integrity number (RIN) higher than 8.0 and the 28S/18S ratio of the qualified RNA ranged from 1.8 to 2.0.

To minimize inter-individual variability, equal amounts of total RNA from three sheep of the same group were pooled into a single biological replicate, and two biological replicates were prepared for each group [43]. One microgram of RNA from each biological replicate was employed as template. To construct the sequencing libraries (a total of four libraries, which includes two biological replicates × two groups), a NEBNext® Ultra™ RNA Library Prep Kit for Illumina® (NEB, USA) was employed following the recommendations of the manufacturer, using index codes for each biological replicate.

The index-coded biological replicates were clustered on a cBot Cluster Generation System with a TruSeq PE Cluster Kit v4-cBot-HS (Illumia, USA) following the manufacturer’s instructions. Then, the libraries were sequenced using an Illumina HiSeq 2500 system, generating paired-end reads.

### Sequenced data processing

The raw reads in FASTQ format were first processed using the FASTX-Toolkit (version: 0.0.13). Here, reads with adapter sequences, low quality 3′ end, containing poly-N, rRNA, sequences shorter than 20 nt and low quality with Q < 20 were eliminated, and the remaining reads were designated as clean reads. We then assessed the resulting clean data in terms of Q30 value, GC-content, and extent of sequence duplication. All subsequent analyses were then based on high quality, clean data. The clean reads from every library were subsequently mapped to the reference genome of sheep (Ovis_aries.Oar_v3.1.74.toplevel.fa, Ensembl release 74) using the TopHat2 (version: 2.0.9). Reads showing a perfect match or a single mismatch were further evaluated and annotated in relation to the reference genome.

### Differential gene expression and functional analysis

Normalization of expression levels of each gene was performed using fragments per kilo base per million mapped reads (FPKM) method. The differentially expressed genes (DEGs) between the LG and the OG groups were detected using the DESeq R package. The false discovery rate (FDR) was subjected to Benjamini and Hochberg correction to adjusted *P*-value. An adjusted *P*-value < 0.05 and a log2 ratio ≥ 1 were used as criteria in selecting significant DEGs. The identified DEGs were functionally annotated using gene ontology (GO), and the Blast2Go program for the categories of biological process, cellular component, and molecular function. The Kyoto Encyclopedia of Genes and Genomes (KEGG) was used to predict metabolic pathways, and statistical enrichment of DEGs for various biological processes and pathways was performed using the KOBAS.

### Experimental validation by quantitative real-time PCR (qRT-PCR)

To assess the repeatability and reproducibility of our gene expression data, which were obtained using RNA-Seq of sheep liver, qRT-PCR was conducted on five randomly selected genes from the significant DEGs using the extracted total RNA (a total of 12 individual liver samples, *n* = 6 for each group) as template. The PrimeScript™ Reverse Transcriptase, D2680A (Takara Biotechnology, China) was employed in first-strand cDNA synthesis. Gene expression levels were assessed using an Applied Biosystems 7500 Fast Real-Time PCR System (Foster City, USA) and a PrimeScript™ RT reagent Kit, RR037A (SYBR Green) (Takara Biotechnology, China). The sequences of the primers used in this study are presented in Additional file 4: Table S3, which were designed using primer premier 5.0. The sheep β-actin (*ACTB*) gene was used as an internal control [44, 45] to normalize mRNA levels, which was consistently stable among the experimental groups and among several other internal control genes examined including glyceraldehyde 3-phosphate dehydrogenase (*GAPDH*), 60S ribosomal protein L19 (*RPL19*) and 60S acidic ribosomal protein P0 (*RPLP0*) using BestKeeper software (Additional file 5: Figure S1). The reaction mixtures were incubated in a 96-well plate at 95 °C for 30 s, followed by 40 cycles of 95 °C for 10 s, 60 °C for 30 s. All measurements were conducted in triplicates. Relative mRNA expression levels were calculated to the Ct value obtained for the housekeeping control gene (*ACTB*) using the 2^−ΔΔCT^ method [46].

### Statistical analyses

Statistical analyses were conducted using SPSS Statistics 17.0 (SPSS, Inc., USA). The results were presented as the mean ± SD. Differences in forage nutritional contents, sheep growth performance, serum parameters and gene expression levels between the two groups were evaluated using two-tailed Student’s *t* test, and significance was established at a *P* < 0.05.

## Additional file

Additional file 1: **Table S1.** The effect of overgrazing on major nutritional indices of herbage. (DOC 33 kb)

Additional file 2: **Table S2.** RNA-Seq data of all identified genes (*n* = 12,860) in the study. (XLSX 1428 kb)

Additional file 3: The detailed description of herbage sample collection. (DOC 28 kb)

Additional file 4: **Table S3.**. The primers used for the qPCR analysis. (DOC 29 kb)

Additional file 5: **Figure S1.**. Analysis of 4 reference genes standard deviation (SD) value using BestKeeper. Reference genes with an SD below 1 are considered stably expressed, and a smaller SD indicates a more stable reference gene. The result showed that *ACTB* is the most stable gene. (TIFF 379 kb)

## Data Availability

The data supporting the results of this article are included within the article and in its additional files.

## Abbreviations

DEGs: Differentially expressed genes;
GO: Gene Ontology
KEGG: Kyoto Encyclopedia of Genes and Genomes
LG: Light grazing
NCBI: National Center for Biotechnology Information
OG: Overgrazing
