# Supplementary material for: Hepatic transcriptome profile of sheep (Ovis aries) in response to overgrazing: novel genes and pathways revealed
Source: BMC Genet. 2019 Jul 4;20:54. doi: 10.1186/s12863-019-0760-x (PMC6610972; doi:10.1186/s12863-019-0760-x)
Supplement: Supplementary file 1 — Table S1. The effect of overgrazing on major nutritional indices of herbage. (DOC 33 kb) [file 12863_2019_760_MOESM1_ESM.doc]

**Table S1 The effects of overgrazing on primary nutritional indices of herbage**

| Item | Groups | |  | |  |
| --- | --- | --- | --- | --- | --- |
|  | LG | OG | |  | *P* value |
| CP (g/kg DM) | 90.4 ± 4.3^b^ | 109.5 ± 6.2^a^ | |  | 0.013 |
| Gross energy (kJ/g DM) | 16.1 ± 0.2^a^ | 14.3 ± 0.1^b^ | |  | 0.009 |
| NFE (g/kg DM) | 48.5 ± 3.9^a^ | 42.3 ± 1.7^b^ | |  | 0.041 |
| NDF (g/kg DM) | 670.3 ± 30.8 | 631.2 ± 58.1 | |  | 0.089 |
| ADF (g/kg DM) | 315.3 ± 19.0 | 337.0 ± 15.0 | |  | 0.162 |
| ADL (g/kg DM) | 149.5 ± 24.3^b^ | 174.6 ± 15.7^a^ | |  | 0.043 |

LG, light grazing; OG, overgrazing.

CP, crude protein; NFE, nitrogen free extract; NDF, neutral detergent fibre; ADF, acid detergent fibre; ADL, acid detergent lignin.

Values within a column not sharing a common superscript letter indicate significant difference at *P* < 0.05. Numbers are means ± SD (n = 3).
