# Supplementary material for: Hepatic transcriptome profile of sheep (Ovis aries) in response to overgrazing: novel genes and pathways revealed
Source: BMC Genet. 2019 Jul 4;20:54. doi: 10.1186/s12863-019-0760-x (PMC6610972; doi:10.1186/s12863-019-0760-x)
Supplement: Supplementary file 3 — The detailed description of herbage sample collection. (DOC 28 kb) [file 12863_2019_760_MOESM3_ESM.doc]

**The detailed description of forage sample collection**

Forage mass was determined, and samples were taken for chemical analysis on July1^st^, August 1^st^ and September 1^st^ respectively. In each paddock one pooled sample was obtained from 3 sub-samples, taken from 1 m^2^ (1×1 m) sized transects. Forage samples included the standing biomass cut to 1 cm stubble height, assuming to be the minimum bite height of a sheep under restricted but not starving conditions. Litter (dead material spread on the ground) was not included into the forage sample. Pooled forage samples were dried in a 60 °C oven for 24 h and ground through a 1 mm screen.
