# Supplementary material for: Hepatic transcriptome profile of sheep (Ovis aries) in response to overgrazing: novel genes and pathways revealed
Source: BMC Genet. 2019 Jul 4;20:54. doi: 10.1186/s12863-019-0760-x (PMC6610972; doi:10.1186/s12863-019-0760-x)
Supplement: Supplementary file 4 — Table S3.. The primers used for the qPCR analysis. (DOC 29 kb) [file 12863_2019_760_MOESM4_ESM.doc]

**Table S3 The primers used for the qPCR analysis**

| Gene name | Primer sequence |
| --- | --- |
| *ATCB* | forward: AGATTGGCCTCGTGCGATTC  reverse: CATGGTTGCTAAGGGCAGGA |
| *TNC* | forward: GAAGTCCACCTCCTGCCAAA  reverse: ACTCCACTCTGTCGCTTGTG |
| *AQP7* | forward: AGGAGAATCGCTTCCCTCACC  reverse: GGTGGATGCAGGGTCACAAAT |
| *IFI27L2* | forward: GCTTCACCGGGGCTGGAAT  reverse: TTGGCCACCGCGGCA |
| *ORM1* | forward: CACCATGGACTGGCTCTCTG  reverse: CCTGGATTGCTCTAGCCGAC |
| *LRRC40* | forward: TGGGTGGACAGGTAGGTAAGTAG  reverse: GCAAATGCAAGCTGTATTCTCCA |
