# Supplementary figures and images for: Hepatic transcriptome profile of sheep (Ovis aries) in response to overgrazing: novel genes and pathways revealed
Source: BMC Genet. 2019 Jul 4;20:54. doi: 10.1186/s12863-019-0760-x (PMC6610972; doi:10.1186/s12863-019-0760-x)

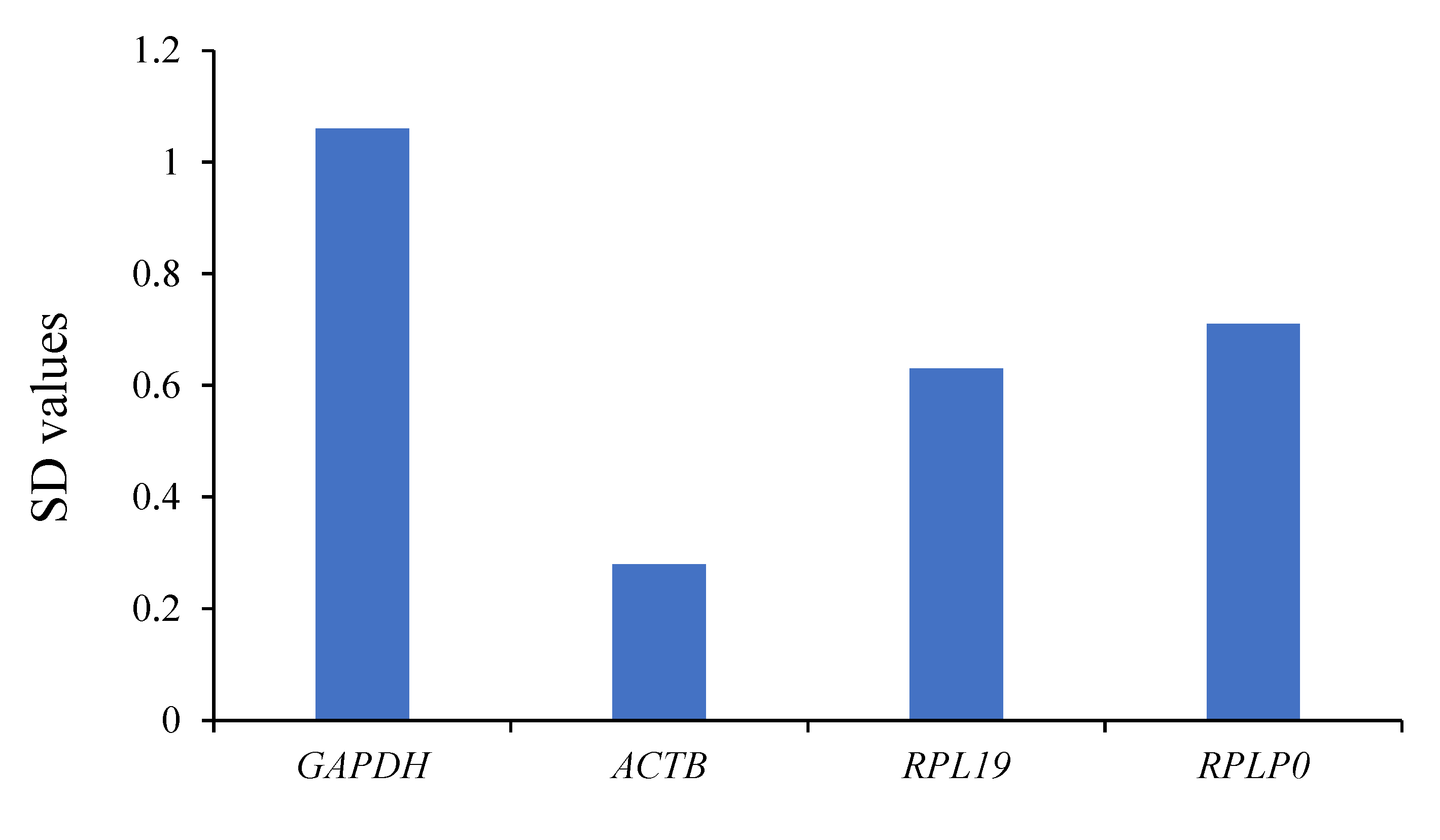

Supplement: Supplementary file 5 — Figure S1.. Analysis of 4 reference genes standard deviation (SD) value using BestKeeper. Reference genes with an SD below 1 are considered stably expressed, and a smaller SD indicates a more stable reference gene. The result showed that ACTB is the most stable gene. (TIFF 379 kb) [file 12863_2019_760_MOESM5_ESM.tiff]
